# Hough Transform-Based Large Dynamic Reflection Coefficient Micro-Motion Target Detection in SAR

**DOI:** 10.3390/s19143227

**Published:** 2019-07-22

**Authors:** Yang Zhou, Daping Bi, Aiguo Shen, Xiaoping Wang, Shuliang Wang

**Affiliations:** College of Electronic Engineering, National University of Defense Technology, Hefei 230037, China

**Keywords:** synthetic aperture radar (SAR), micro-Doppler effect, detection algorithm, parameter estimation, TF analysis, Hough transform, large dynamic reflection coefficient

## Abstract

Special phase modulation of SAR echoes resulted from target rotation or vibration, is a phenomenon called the micro-Doppler (m-D) effect. Such an effect offers favorable information for micro-motion (MM) target detection, thereby improving the performance of the synthetic aperture radar (SAR) system. However, when there are MM targets with large differences in reflection coefficient, the weak reflection components will be difficult to be detected. To find a solution to this problem, we propose a novel algorithm. First, we extract and detect the strongest reflection component. By removing the strongest reflection component from the original azimuth echo one by one, we realize the detection of reflection components sequentially, from the strongest to the weakest. Our algorithm applies to detecting MM targets with different reflection coefficients and has high precision of parameter estimation. The results of simulation and field experiments verify the advantages of the algorithm.

## 1. Introduction

In the detection area, there exist a type of micro-motion (MM) targets like rotating antennas and vibrating vehicle engines, which cause complex nonlinear phase modulation of SAR echoes. Such a phenomenon is termed micro-Doppler (m-D) effect [1]. This effect destroys SAR echo signal characteristics that SAR imaging algorithms rely on. Consequently, poor SAR azimuth focusing phenomenon (such as ghosts and fences) appears [2,3]. However, the nonlinear modulation phase of SAR echoes contains the feature information of the MM targets. By mining the content of the information, the structural features and motion features of MM targets become available [4,5,6,7]. This is of great significance in the field of battlefield reconnaissance and surveillance, target identification [8], and precision guidance, so it has become a research hotspot in the field of SAR.

At present, there has been a lot of research into the feature extraction of MM targets. The mainstream method of MM target detection is dealing with the time-frequency (TF) diagram of the echo [9,10,11,12] and improve the distribution structure of m-D signals in primitive domain by seeking various transformations (for example, Hough transform (HT) [13,14,15], inverse Radon transform (IRT) [16] and wavelet transform [17]), so as to extract signal features better. In view of MM features of vibration and rotation, MM target azimuth echoes are generally modeled as sinusoidal frequency modulated (SFM) signals [18,19]. Namely, an SFM signal will appear as a sinusoidal curve on the TF distribution. Therefore, the MM features can be extracted by detecting sinusoidal curves in TF distribution. However, in a real scenario, the azimuth echoes are usually mixed with multiple MM target components, and the reflection coefficient of each MM target is usually different. When there are both weak and strong reflection components in the scenario, the weak ones are often masked by the strong ones and become difficult to be detected. Therefore, some algorithms will fail to extract the weak MM targets, such as in [10,12,15]. So far, there are few effective algorithms for large dynamic reflection coefficient MM target detection and parameter estimation. Military sensitive targets (such as rotating antenna, helicopter rotor) with weak reflection coefficients cannot be detected timely. This constitutes a serious hidden danger.

To solve this problem, we propose a novel algorithm to detect MM targets with large dynamic reflection coefficient. Firstly, the MM target azimuth echo is analyzed in TF domain, and the TF curve of the strongest component in the TF distribution is extracted. Then, the HT is applied to the TF curve to estimate the parameters of the strongest reflection component. Then, we removed the strongest component from the original echo to highlight the weak reflection components. The above process is repeated and the strong targets are removed one by one until all the MM targets are detected. In this algorithm, we replace IRT [16] with HT to detect MM targets, because the IRT algorithm only applies to centered SFM signals. If the signal is a non-centered SFM signal, the result of IRT will defocus [20]. Since the analysis formula of non-centered sinusoidal curve in TF domain can be written, the detection of MM target with non-centered SFM echo signal can be solved by HT.

The remainder of this study is organized as follows. We present the model of rotating targets in Section 2. In Section 3, we describe the principles and procedures of the algorithm. We conduct simulation and field experimental data research in Section 4. Section 5 gives the conclusion.

## 2. Signal Model

### 2.1. Rotating-Target Geometry

**Figure 1:**
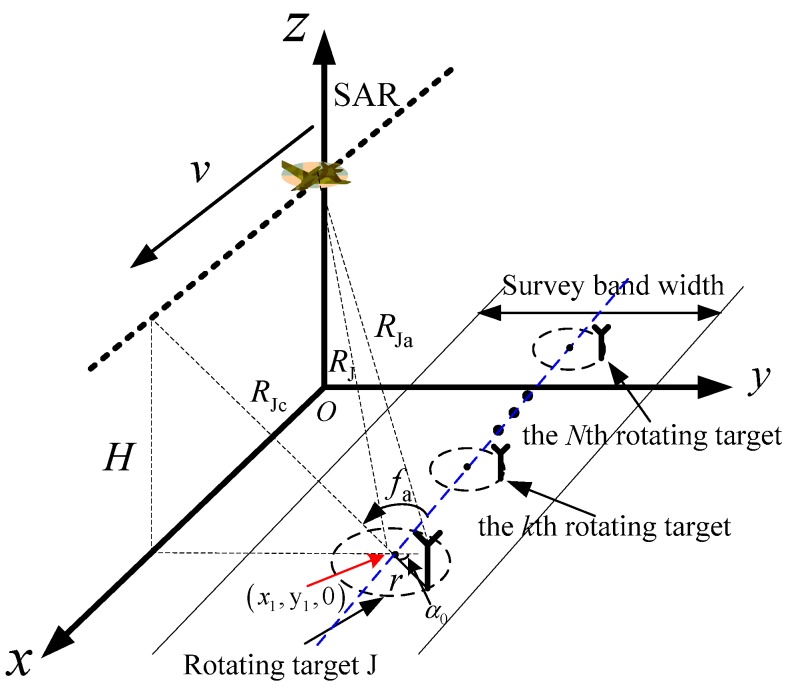
Radar-target geometry.

In Figure 1, SAR is equipped on the aircraft that moves along x-axis with velocity v. The altitude of the aircraft is H. In the ground, the rotational radius of rotating target J is r and its frequency is $f_{a}$. The coordinate of rotating center is $\left(x_{1},y_{1},0\right)$. The distance from SAR to the rotating center is $R_{J}=\sqrt{x_{1}^{2}+y_{1}^{2}+H^{2}}$ when the slow time is $t_{a}=0$. The initial phase of J is $\alpha_{0}$. J’s coordinates are $\left(x_{1}+r\sin\left(2\pi f_{a}t_{a}+\alpha_{0}\right),y_{1}+r\cos\left(2\pi f_{a}t_{a}+\alpha_{0}\right),0\right)$. Therefore, the distance from SAR to J is

(1) $$\begin{array}{ll} R_{\mathrm{Ja}}\left(t_{a}\right) & =\sqrt{{\left[x_{1}+r\sin\left(2\pi f_{a}t_{a}+\alpha_{0}\right)-vt_{a}\right]}^{2}+{\left[y_{1}+r\cos\left(2\pi f_{a}t_{a}+\alpha_{0}\right)\right]}^{2}+H^{2}}\\ & \approx R_{J}-\frac{x_{1}v}{R_{J}}t_{a}+\frac{v^{2}}{2R_{J}}t_{a}^{2}+\frac{r^{2}}{2R_{J}}+r_{0}\cos\left(2\pi f_{a}t_{a}+\varphi_{0}\right) \end{array}$$

where $\varphi_{0}=\alpha_{0}-\arctan\left[\left(x_{1}-vt_{a}\right)/y_{1}\right]$ and $r_{0}=r\sqrt{{\left(x_{1}-vt_{a}\right)}^{2}+y_{1}^{2}}/R_{J}$. The approximate condition of Equation (1) is when $R_{J}\gg r,x_{1}$. When the distance between J and the track of the aircraft is far enough, the variation of $\left(x_{1}-vt_{a}\right)$ is very small relative to $y_{1}$, $\varphi_{0}$ and $r_{0}$ are close to constant, and $r_{0}$ is named to be the effective radius.

### 2.2. SAR Azimuth Echo

The signal transmitted by SAR is expressed as

(2) $$s_{0}\left(t_{r}\right)={\mathrm{rect}}_{T_{p}}\left(t_{r}\right)\exp\left[j2\pi \left(f_{0}t+\mu_{r}t_{r}^{2}/2\right)\right]$$

where ${\mathrm{rect}}_{T_{p}}\left(\cdot \right)$ denotes the rectangular window function and its width is $T_{p}$. $t_{r}$ denotes the fast time, $T_{p}$ the pulse width, $\mu_{r}$ the chirp rate, and $f_{0}$ the carrier frequency.

The base-band signal of J’s echo received by SAR is

(3) $$s_{J}\left(t_{r},t_{a}\right)=\sigma_{J}\cdot {\mathrm{rect}}_{T_{P}}\left[t_{r}-\frac{2R_{\mathrm{Ja}}\left(t_{a}\right)}{c}\right]\exp\{j\pi \mu_{r}{\left[t_{r}-\frac{2R_{\mathrm{Ja}}\left(t_{a}\right)}{c}\right]}^{2}\}\exp\left[-j\frac{4\pi }{\lambda }R_{\mathrm{Ja}}\left(t_{a}\right)\right]$$

where $\sigma_{J}$ denotes the reflection coefficient of J. In Equation (3), the first exponential term represents the phase change caused by the delay of the signal, and the second exponential term represents the phase change caused by the change of SAR azimuth position. By performing range compression and range migration correction, we obtain

(4) $$s_{\mathrm{Jr}}\left(t_{r},t_{a}\right)=\sigma_{J}\cdot \left(1-\left|t_{\mathrm{Jr}}^{*}\right|/T_{P}\right)\cdot \mathrm{sinc}\left[\pi \mu_{r}t_{\mathrm{Jr}}^{*}\left(T_{p}-\left|t_{\mathrm{Jr}}^{*}\right|\right)\right]\exp\left[-j\frac{4\pi }{\lambda }R_{\mathrm{Ja}}\left(t_{a}\right)\right]$$

where $t_{\mathrm{Jr}}^{*}=t_{r}-2R_{\mathrm{Jc}}/c$, $R_{\mathrm{Jc}}=\sqrt{y_{1}^{2}+H^{2}}$ denotes the distance from the rotating center to the track. In Equation (4), we can see that the energy of each pulse in the range focuses on a peak after range compression. Extract these peaks in each pulse (that is, set $t_{r}=2R_{\mathrm{Jc}}/c$) and substitute the result of Equation (1) into Equation (4), and the signal expression is

(5) $$s_{\mathrm{Jr}}\left(t_{a}\right)=\sigma_{J}\cdot \exp\{-j\frac{2\pi }{\lambda }[2R_{J}-\frac{2x_{1}v}{R_{J}}t_{a}+\frac{v^{2}}{R_{J}}t_{a}^{2}+\frac{r^{2}}{R_{J}}+2r_{0}\cos\left(2\pi f_{a}t_{a}+\varphi_{0}\right)]\}$$

After multiplying $\exp\left(j2\pi v^{2}t_{a}^{2}/\lambda R_{J}\right)$ to remove the chirp term in Equation (5), known as dechirp processing, we obtain the signal expression

(6) $$s_{\mathrm{Jc}}\left(t_{a}\right)=\sigma \cdot \exp\left[j2\pi Ft_{a}-j\frac{A_{\omega }}{f_{a}}\cos\left(2\pi f_{a}t_{a}+\varphi_{0}\right)\right]$$

where $s_{\mathrm{Jc}}\left(t_{a}\right)$ is referred as the rotating target azimuth echo, $F=2vx_{1}/\lambda R_{\mathrm{Jc}}$ denotes the azimuth center frequency, σ denotes a complex constant, and $A_{\omega }=4\pi r_{0}f_{a}/\lambda$ denotes the maximum m-D amplitude. Deriving the phase of $s_{\mathrm{Jc}}\left(t_{a}\right)$ to the time $t_{a}$, we obtain the instant Doppler frequency expression:

(7) $$f_{d}\left(t_{a}\right)=A_{\omega }\sin\left(2\pi f_{a}t_{a}+\varphi_{0}\right)+F$$

It can be seen that $s_{\mathrm{Jc}}\left(t_{a}\right)$ is an SFM signal. Without losing generality, when there are *K* rotating targets in a single range cell (see Figure 1), the expression of azimuth echo can be expressed to be

(8) $$s\left(t_{a}\right)=\sum_{k=1}^{K}\sigma^{\left(k\right)}\cdot \exp\left[j2\pi F^{\left(k\right)}t_{a}-j\frac{A_{\omega }^{\left(k\right)}}{f_{a}^{\left(k\right)}}\cos\left(2\pi f_{a}^{\left(k\right)}t_{a}+\varphi_{0}^{\left(k\right)}\right)\right]$$

where ${\left(\cdot \right)}^{\left(k\right)}$ denotes the *k*th MM target parameter, for example, $\sigma^{\left(k\right)}$ denotes the *k*th MM target reflection coefficient. Thus, the azimuth echo signal $s\left(t_{a}\right)$ consists of multiple SFM signals with different parameters.

### 2.3. Masking Phenomenon of Weak MM Targets

Nowadays, the prevalent methods of MM target detection use TF transform to obtain TF distribution, and to map TF curve to parameter space by means of various transform. However, these methods merely apply to the detection of a single MM target or several MM targets with similar reflection coefficients. In practice, the reflection coefficients of each MM target are often different. If there are MM targets with large difference in reflection coefficients in the echo, the TF components of the weak targets will probably be masked by strong targets in TF distribution. Usually, only TF components of the strong targets can be observed, while the TF components of the weak targets cannot be observed. This is the masking phenomenon of weak MM targets.

**Figure 2:**
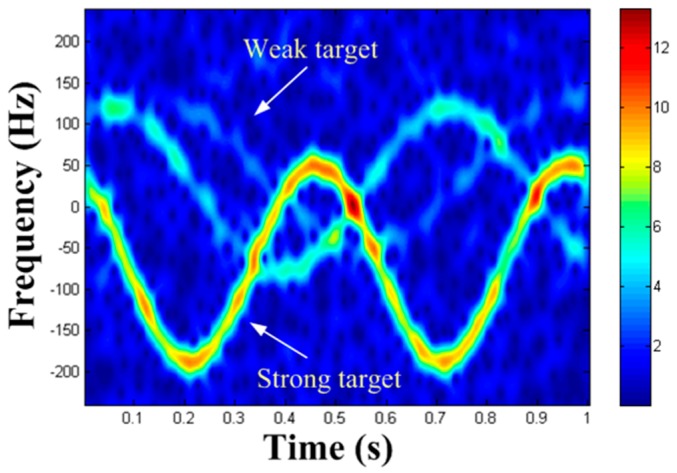
Time-frequency (TF) distribution of three micro-motion (MM) targets with different reflection coefficients.

In order to visualize the masking phenomenon, Figure 2 demonstrates a TF distribution of azimuth echo mixed with three MM targets with different reflection coefficients. In Figure 2, the TF component of the strong target is clearly visible, while the TF component of the weak target is almost impossible to observe. Therefore, many traditional detection methods based on TF analysis are no longer applicable to the detection of MM targets with large dynamic reflection coefficients.

## 3. Detection Algorithm for Large Dynamic Reflection Coefficient MM Targets

### 3.1. Principles

Section 2.2 points out that the azimuth signal of MM target can be modeled as SFM signal, so this paper achieves the detection of the MM target by detecting the SFM signal from SAR azimuth echo. Thereby, we first extract SAR azimuth echo $s\left(t_{a}\right)$ from the raw data. The TF distribution of SFM signal is in form of sinusoidal curve and HT has good parameter estimation performance for sinusoidal curve, so we can use HT to detect SFM signal. 

The essence of HT is to turn the feature curve from the image domain to the parameter domain via the curve’s analysis formula. If a curve satisfying the analysis formula exists, a peak will emerge. By searching for the peak in the parameter domain, we can realize the detection and parameter estimation of the detected curve. From Equation (8), the analysis formula of each TF curve of MM targets is as follows:

(9) $$f_{d}\left(t_{a}\right)=A_{\omega }^{\left(k\right)}\sin\left(2\pi f_{a}^{\left(k\right)}t_{a}+\varphi_{0}^{\left(k\right)}\right)+F^{\left(k\right)}$$

According to Equation (9), we can establish the parameter space $K=\left(A_{k},\varphi_{k},F_{k},f_{k}\right)$. The computational complexity of HT will increase with the growth of the parameter space dimension [21]. Hence, it is necessary to decrease the dimension of parameter space or optimize the algorithm. The MM frequency can be acquired by the autocorrelation method [22] with ease. Thus, the parameter space is lessened to $K=\left(A_{k},\varphi_{k},F_{k}\right)$. This greatly reduces the search amount of HT compared with [14]. If the sampling rate is $f_{s}$, the maximum value at the sampling point of the autocorrelation result is $n_{1}$, and the secondary maximum value at the sampling point is $n_{2}$, then the estimated value of MM period is given by

(10) $${\hat{T}}_{a}={1/\hat{f}}_{a}=\left|n_{1}-n_{2}\right|/f_{s}$$

Next, we extract the strongest TF curve from the TF distribution of azimuth echo $s\left(t_{a}\right)$ and carry out HT on the TF curve to get a 3D cumulative array $\mathrm{Acc}\left(A_{k},\varphi_{k},F_{k}\right)$. Then, we search the peak in $\mathrm{Acc}\left(A_{k},\varphi_{k},F_{k}\right)$ to realize the strongest target detection. However, a problem arises in searching peak in the cumulative array $\mathrm{Acc}\left(A_{k},\varphi_{k},F_{k}\right)$. Many sources of error (such as strong noise) effect the computation of the parameter space $K=\left(A_{k},\varphi_{k},F_{k}\right)$ so that in general many array locations in the vicinity of the ideal peak in $\mathrm{Acc}\left(A_{k},\varphi_{k},F_{k}\right)$ are incremented instead of the peak itself. That is to say, HT can lead to numerous false peaks near the ideal peak. In general, the values of false peaks are smaller than that of the ideal peak. So, we search $\mathrm{Acc}\left(A_{k},\varphi_{k},F_{k}\right)$ for the maximum in the parameter space. And the ideal peak is the one that corresponds to the maximum in the parameter space. By using this method, we obtain the strongest MM target estimated value as $\left({\hat{A}}_{\omega }^{\left(k\right)},{\hat{\varphi }}_{0}^{\left(k\right)},{\hat{F}}^{\left(k\right)},{\hat{f}}_{a}^{\left(k\right)}\right)$.

In order to simplify weak reflection component detection, we can remove the strongest reflection component from the original SAR azimuth echo $s\left(t_{a}\right)$. The specific steps of removal are as follows. First, construct signal $s_{d}\left(t_{a}\right)=\exp[-j2\pi {\hat{F}}^{\left(k\right)}t+j\frac{{\hat{A}}_{\omega }^{\left(k\right)}}{{\hat{f}}_{a}^{\left(k\right)}}\cos\left(2\pi {\hat{f}}_{a}^{\left(k\right)}t+{\hat{\varphi }}_{0}^{\left(k\right)}\right)]$. Multiply the azimuth echo $s\left(t_{a}\right)$ by $s_{d}\left(t_{a}\right)$, that is $s_{f}\left(t_{a}\right)=s\left(t_{a}\right)\cdot s_{d}\left(t_{a}\right)$. This step is to make the phase of the strongest reflection component signal zero. Then, let $s_{f}\left(t_{a}\right)$ pass a high-pass filter h(t) whose cut-off frequency is close to zero to obtain a filtered output $w\left(t_{a}\right)=s_{f}\left(t_{a}\right)⊗h\left(t_{a}\right)$, where ‘⊗’ represents a convolution operation. In this step, we manage to remove this strongest component. Next, multiply the filtered output $w\left(t_{a}\right)$ by the conjugate of $s_{d}\left(t_{a}\right)$ to obtain the signal $s_{res}\left(t_{a}\right)=w\left(t_{a}\right)\cdot s_{d}^{*}\left(t_{a}\right)$. Here, $s_{res}\left(t_{a}\right)$ is the azimuth echo whose strongest component is removed. This step achieves the recovery of the phases of the rest components. Then, set $s\left(t_{a}\right)=s_{res}\left(t_{a}\right)$. After the strongest reflection component is removed, weak refection components become easier to be observed in the TF distribution.

Finally, the above detection steps are repeated, and the strong reflection components in the azimuth echo are removed one by one until there is no MM component in the echo. This is why the algorithm can achieve large dynamic reflection coefficient MM target detection.

### 3.2. TF Curve Extraction

**Figure 3:**
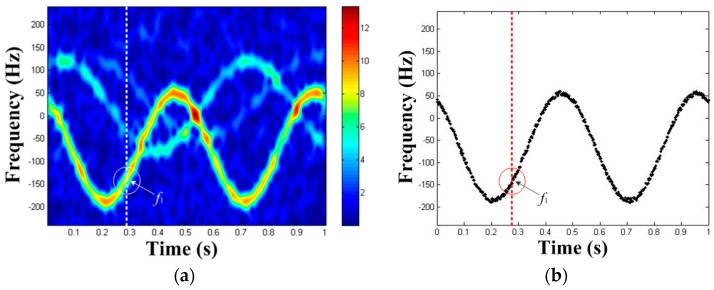
The schematic diagram of TF curve extraction: (**a**) TF curve extraction process, (**b**) TF curve extraction result.

In TF distribution, all bands of spectrum usually have a certain width (see Figure 2). Hence, the HT cannot be used directly. If the HT is directly performed in TF distribution, it will not only consume too much time, but also defocus the peak in the parameter domain and reduce the precision of parameter estimation. Hence, TF curve extraction is needed. Here, we present a method for extracting the strongest TF curve in TF distribution. The main operation is to extract the frequency of the TF distribution at each moment corresponding to the maximum amplitude of the frequency sequence. And we view the extracted frequency as the frequency value of the TF curve at that time. The schematic diagram of TF curve extraction is shown in Figure 3, where the point that is circled in Figure 3a indicates the instant frequency $f_{1}$ extracted at the time of 0.29 s, and Figure 3b indicates the TF curve extraction result. 

### 3.3. Algorithm Steps

**Figure 4:**
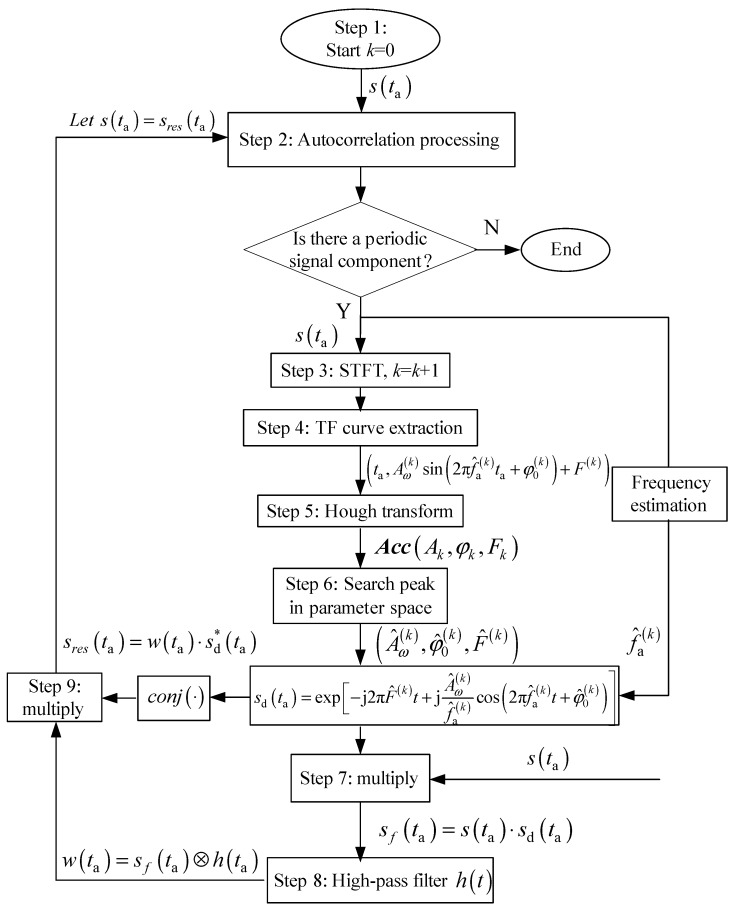
The algorithm flow chart.

The main procedures of the algorithm for large dynamic reflection coefficient MM targets are as follows (the flow chart can be seen in Figure 4):

Step 1: Extract SAR azimuth signal $s\left(t_{a}\right)$. Set k=0.

Step 2: Perform the autocorrelation in $s\left(t_{a}\right)$ and judge whether there is a periodic signal component. If it does exist, get the frequency estimation ${\hat{f}}_{a}^{\left(k\right)}$ of the periodic component and set k=k+1. Go to step 3. If it does not exist, stop detecting. In general, when there are multiple periodic signal components in $s\left(t_{a}\right)$, the autocorrelation method can only obtain frequency estimation of the strongest component [22].

Step 3: Obtain TF distribution of $s\left(t_{a}\right)$ using appropriate TF analysis method. STFT has good TF resolution and no cross-terms in TF domain. For this reason, we select STFT.

Step 4: Utilize the method in Section 3.2 to extract TF curve. If a MM component exists, its TF curve coordinates will be shown as $\left(t_{a},A_{\omega }^{\left(k\right)}\sin\left(2\pi {\hat{f}}_{a}^{\left(k\right)}t_{a}+\varphi_{0}^{\left(k\right)}\right)+F^{\left(k\right)}\right)$.

Step 5: Establish parameter space $K=\left(A_{k},\varphi_{k},F_{k}\right)$ and decide on the proper step size in search and search scope. Carry out the TF curve HT to get a 3D cumulative array $\mathrm{Acc}\left(A_{k},\varphi_{k},F_{k}\right)$.

Step 6: Search the ideal peak in $\mathrm{Acc}\left(A_{k},\varphi_{k},F_{k}\right)$ using maximum search method. In Cartesian coordinates, the *x*-axis of this peak signifies the estimated value of the maximal m-D frequency ${\hat{A}}_{\omega }^{\left(k\right)}$; the *y*-axis of this peak signifies the estimated value of the initial phase ${\hat{\varphi }}_{0}^{\left(k\right)}$; and the *z*-axis of this peak signifies the estimated value of the azimuth center frequency ${\hat{F}}^{\left(k\right)}$. Then, construct signal $s_{d}\left(t_{a}\right)=\exp\left[-j2\pi {\hat{F}}^{\left(k\right)}t_{a}+j\frac{{\hat{A}}_{\omega }^{\left(k\right)}}{{\hat{f}}_{a}^{\left(k\right)}}\cos\left(2\pi {\hat{f}}_{a}^{\left(k\right)}t_{a}+{\hat{\varphi }}_{0}^{\left(k\right)}\right)\right]$.

Step 7: Set $s_{f}\left(t_{a}\right)=s\left(t_{a}\right)\cdot s_{d}\left(t_{a}\right)$.

Step 8: Let $s_{f}\left(t\right)$ pass a high-pass filter h(t) with a cut-off frequency close to zero to get the filtered output: $w\left(t_{a}\right)=s_{f}\left(t_{a}\right)⊗h\left(t_{a}\right)$.

Step 9: Set $s_{res}\left(t_{a}\right)=w\left(t_{a}\right)\cdot s_{d}^{*}\left(t_{a}\right)$. At this point, the strongest component has been removed from $s\left(t_{a}\right)$.

Step 10: Set $s\left(t_{a}\right)=s_{res}\left(t_{a}\right)$. Repeat steps 2–10 until all MM targets are detected.

## 4. Simulation and Field Experiment Data Research

### 4.1. Algorithm Step Simulations

To prove the validity of the proposed algorithm, we carry out the following simulations. Figure 1 is the scenario of the simulation. In the simulation, the altitude of the aircraft is 6000 m, the flight velocity is 200 m/s. The carrier frequency, PRF and synthetic aperture time of SAR are 10 GHz, 480 Hz, and 1 s, respectively. Set three rotation targets T1, T2 and T3 in a single range cell. Their parameters are in Table 1. According to the motion state and position of T1, T2 and T3, the echo signal received by SAR is simulated by computer as the raw data. According to $A_{\omega }=4\pi r_{0}f_{a}/\lambda$ and $F=2vx_{1}/\lambda R_{\mathrm{Jc}}$, the theoretical values of $A_{\omega }$ of T1, T2 and T3 come out to be 125.6 Hz, 100.5 Hz and 90.4 Hz, respectively, and their theoretical values of F are −70.5 Hz, 20 Hz and 40 Hz, respectively. Presume the azimuth echo of the rotating targets are mixed with Gaussian white noise $n\left(t_{a}\right)$, the received azimuth echo can be shown as

(11) $$x\left(t_{a}\right)=s\left(t_{a}\right)+n\left(t_{a}\right)$$

Set the signal-to-noise ratio (SNR) as −2 dB.

**Figure 5:**
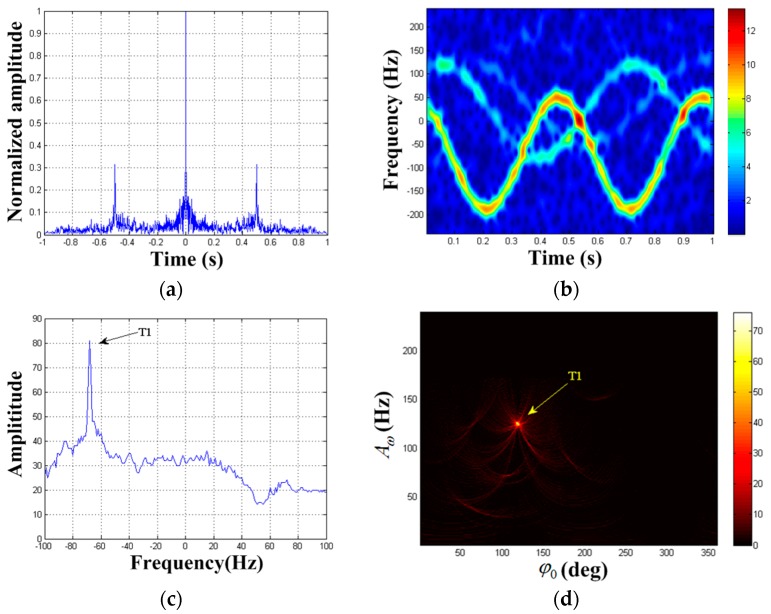
Detection results of $x\left(t_{a}\right)$. (**a**) Autocorrelation result of $x\left(t_{a}\right)$; (**b**) TF distribution result of $x\left(t_{a}\right)$; (**c**) $F_{1}$-domain; (**d**) $\left\{A_{1},\varphi_{1}\right\}$ domain when $F_{1}=-69\;\mathrm{Hz}$.

The autocorrelation result of $x\left(t_{a}\right)$ is shown in Figure 5a. It is found that periodic component is present. We can get the frequency estimation of periodic component as ${\hat{f}}_{a}^{\left(1\right)}=2\;\mathrm{Hz}$. STFT is performed on $x\left(t_{a}\right)$ (the Kaiser window’s width is 45, the same below), and the TF distribution result can be seen in Figure 5b. The strong reflection component is clearly visible while the weak reflection component is almost impossible to observe. This is the masking phenomenon of weak MM target. Extract the TF curve according to Section 3.2, and establish the parameter space $K=\left(A_{1},\varphi_{1},F_{1}\right)$. Set the maximum Doppler frequency search scope is 0∼240 Hz, the center frequency search scope is −100∼100 Hz, the two frequency step size in search is 1 Hz, the phase search scope is $0∼360^{{}^{\circ}}$, and the phase step size in search is $1^{{}^{\circ}}$. The HT is carried out on the extracted TF curve and $\mathrm{Acc}\left(A_{1},\varphi_{1},F_{1}\right)$ is obtained. The results are illustrated in Figure 5c,d. Figure 5c denotes the HT result versus center frequency F within the search scope, thereby obtaining ${\hat{F}}^{\left(1\right)}=-69\;\mathrm{Hz}$. Hence, the image of $\mathrm{Acc}\left(A_{1},\varphi_{1},-69\mathrm{Hz}\right)$ is drawn in Figure 5d, where the ordinate indicates the maximum m-D amplitude and the abscissa indicates the initial phase value. We can see that the rotating target has formed a peak in Figure 5d. By seeking the peak in $\left\{A_{1},\varphi_{1}\right\}$ domain, we get the estimated value of the first target parameter as $\left({\hat{A}}_{\omega }^{\left(1\right)},{\hat{\varphi }}_{0}^{\left(1\right)}\right)=\left(125\mathrm{Hz},120^{{}^{\circ}}\right)$.

**Figure 6:**
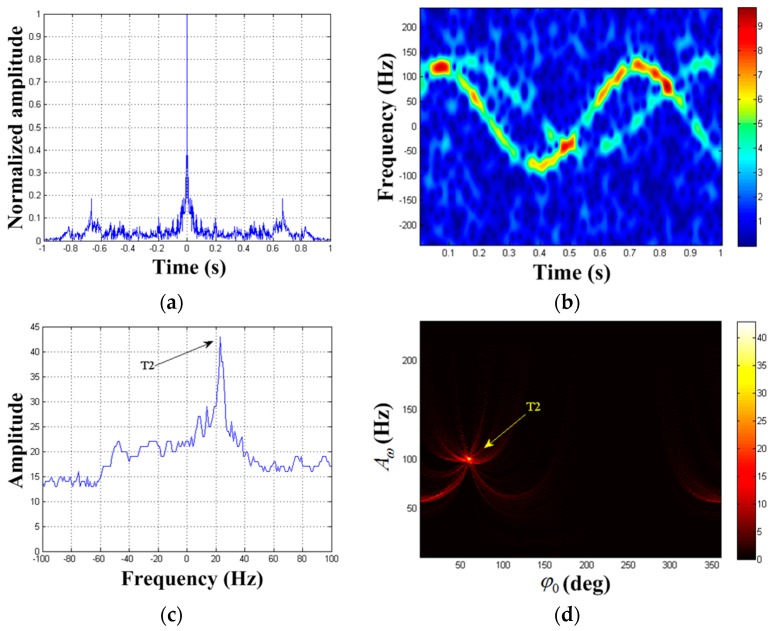
Detection results of $x_{1}\left(t_{a}\right)$. (**a**) Autocorrelation result of $x_{1}\left(t_{a}\right)$; (**b**) TF distribution result of $x_{1}\left(t_{a}\right)$; (**c**) $F_{2}$-domain; (**d**) $\left\{A_{2},\varphi_{2}\right\}$ domain when $F_{2}=21\;\mathrm{Hz}$.

According to steps 7–9 in Section 3.3, the first MM component is removed from $x\left(t_{a}\right)$ and we can get signal $x_{1}\left(t_{a}\right)$. After performing $x_{1}\left(t_{a}\right)$ autocorrelation processing, the result is reflected in Figure 6a. We find the presence of periodic signal component. We can get the frequency estimation of the second periodic component as ${\hat{f}}_{a}^{\left(2\right)}=1.5\;\mathrm{Hz}$. STFT result of $x_{1}\left(t_{a}\right)$ is illustrated in Figure 6b and the first component has been removed. After performing HT on the extracted TF curve, the results are reflected in Figure 6c,d. Similarly, we get the estimated values of the second target parameter as $\left({\hat{A}}_{\omega }^{\left(2\right)},{\hat{\varphi }}_{0}^{\left(2\right)},{\hat{F}}^{\left(2\right)}\right)=\left(101\;\mathrm{Hz},60^{{}^{\circ}},21\;\mathrm{Hz}\right)$.

**Figure 7:**
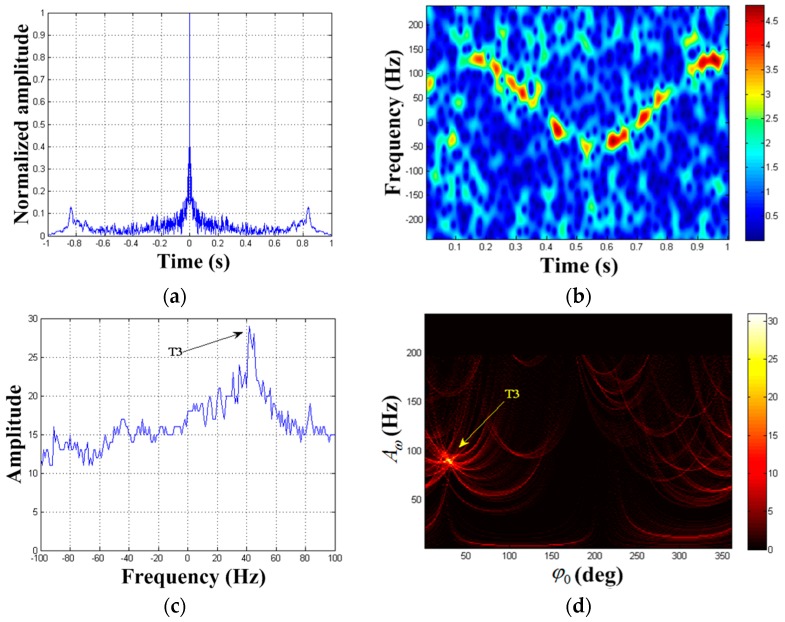
Detection result of $x_{2}\left(t_{a}\right)$. (**a**) Autocorrelation result of $x_{2}\left(t_{a}\right)$; (**b**) TF distribution result of $x_{2}\left(t_{a}\right)$; (**c**) $F_{3}$-domain; (**d**) $\left\{A_{3},\varphi_{3}\right\}$ domain when $F_{3}=43\;\mathrm{Hz}$.

After the second MM component is removed from $x_{1}\left(t_{a}\right)$, we can get signal $x_{2}\left(t_{a}\right)$. The autocorrelation result of $x_{2}\left(t_{a}\right)$ is shown in Figure 7a. We find that periodic signal component is still present. The frequency estimation of the third periodic component is ${\hat{f}}_{a}^{\left(3\right)}=1.2\;\mathrm{Hz}$. Figure 7b demonstrates the STFT result of $x_{2}\left(t_{a}\right)$. We can see that the second component is successfully removed and the weak reflection component is clearly displayed in the TF distribution. Elimination of strong targets prove to be a useful method in weak target enhancement. The results of HT are illustrated in Figure 7c,d. Hence, we can get the estimated values of the third target parameter as $\left({\hat{A}}_{\omega }^{\left(3\right)},{\hat{\varphi }}_{0}^{\left(3\right)},{\hat{F}}^{\left(3\right)}\right)=$$\left(89\;\mathrm{Hz},29^{{}^{\circ}},43\;\mathrm{Hz}\right)$.

**Figure 8:**
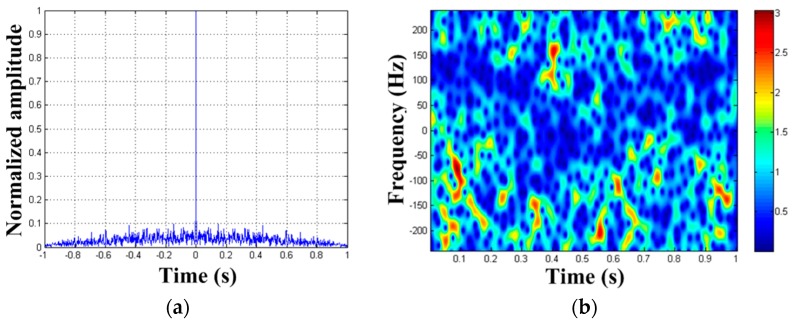
Detection result of $x_{3}\left(t_{a}\right)$. (**a**) Autocorrelation result of $x_{3}\left(t_{a}\right)$; (**b**) TF distribution result of $x_{3}\left(t_{a}\right)$.

After the third MM component is removed from $x_{2}\left(t_{a}\right)$, we can get signal $x_{3}\left(t_{a}\right)$. The autocorrelation result and STFT result of $x_{3}\left(t_{a}\right)$ are shown in Figure 8. We cannot find any periodic signal component from the results, so we stop detecting. So far, all the three rotating targets set in the simulation have been detected. We make a comparison between the estimated values of the parameters and their theoretical values. The results are shown in Table 2. We find that the estimated values of parameters are in accordance with the theoretical values. The final processed results show that we successfully achieve the detection of large dynamic reflection coefficient MM targets. Therefore, it proves the correctness of our algorithm.

**Figure 9:**
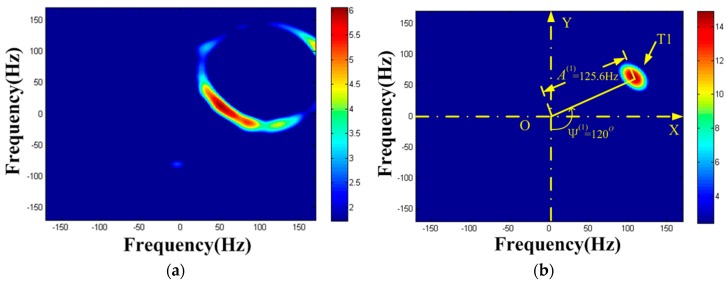
IRT result. (**a**) When the rotational center coordinate of T1 is (−53 m,8000 m,0). (**b**) When the rotational center coordinate of T1 is (0,8000 m,0).

To compare our algorithm with the one proposed in [16], we use the IRT algorithm to detect the MM target T1. Figure 9a demonstrates the IRT result. The IRT result is defocused, so we fail to obtain any information about T1. This is because the azimuth echo generated by T1 is a non-centered SFM signal ($F^{\left(1\right)}=-70.5\;\mathrm{Hz}\neq 0$). If the rotational center coordinates of T1 is changed to (0,8000 m,0), the detection result of T1 is shown in Figure 9b. It can be seen that the IRT algorithm can accurately detect T1, since the azimuth echo generated by T1 is a centered SFM signal ($F^{\left(1\right)}=0$) this time. Similarly, the center frequencies of T2 and T3 are not zero, so the IRT algorithm cannot detect T2, T3. Therefore, our algorithm has a broader scope of applications for MM target detection compared with the IRT algorithm. 

**Figure 10:**
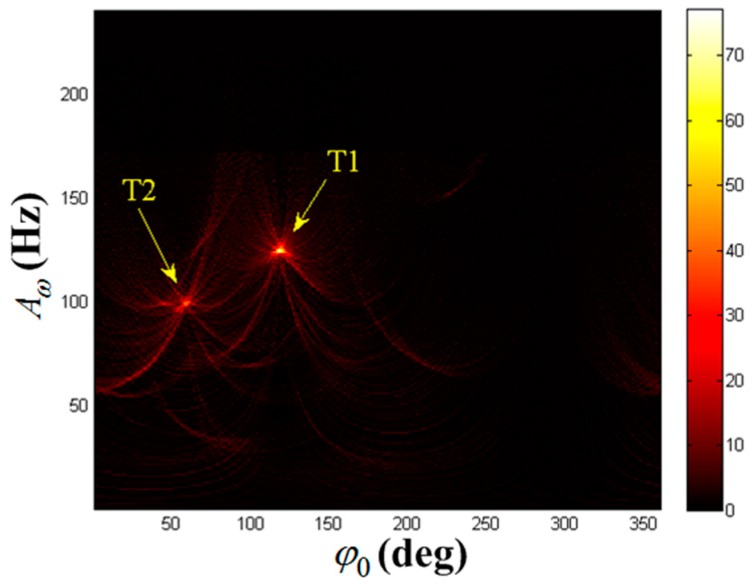
Detection result in [15].

The algorithm in [15] is used to detect T1, T2 and T3, and the detection results are shown in Figure 10. It can be seen that the algorithm in [15] can accurately detect T1 and T2, but it is not possible to obtain any information about T3. This is because T3 is too weak, and the TF curve of T3 cannot be extracted by the algorithm in [15]. Therefore, compared with the algorithm in [15], the algorithm in this paper is able to detect the MM targets with larger reflection coefficient difference.

### 4.2. Algorithm Performance Analysis

#### 4.2.1. Computational Load Analysis

The proposed algorithm principally searches for matching parameters, so the computational complexity primarily rests with the step size in search and search scope of the parameters. Suppose the upper limit of the maximal m-D frequency is $A_{M}$ and the frequency step size in search is $\Delta f_{1}$, the maximal m-D frequency is required $A_{M}/\Delta f_{1}$ search times. Similarly, the center frequency F is required to be searched for $F/\Delta f_{2}$ times, and the initial phase is required to be searched 360/Δφ times, where $\Delta f_{2}$ denotes center frequency while Δφ denotes initial phase step size in search. Therefore, the overall computational complexity is approximately

(12) $$C=\frac{A_{M}}{\Delta f_{1}}\cdot \frac{F}{\Delta f_{2}}\cdot \frac{360}{\Delta \varphi_{1}}\cdot N$$

where N denotes the point number that makes up the TF curve. In Equation (12), *C* is proportional to $A_{M}$ and F, and inversely proportional to $\Delta f_{1}$, $\Delta f_{2}$ and Δφ. In Section 4.1, the simulation software is MATLAB R2014a. The simulation platform is a 32-bit operating system HP computer with Inter Core i3 CPU and with 2 GB of RAM. The operation time required to obtain the HT results in Figure 5, Figure 6, and Figure 7 is 10 s, 12 s, and 11 s, respectively. The time needed is a little long because of 3D parameter searching. If we use the algorithm proposed in [14] to detect T1, T2 and T3, the search range of MM frequency is set as 0∼2 Hz and the search step size is 0.1 Hz, and the other parameter search range and search step size are consistent with the algorithm in this paper. Then, the operation time required to obtain the detection result of T1, T2 and T3 is 205 s, 243 s and 221 s, respectively. It can be seen that the operation time is much longer than that of the algorithm in this paper, which is because the 4D parameter searching is carried out in [14]. To cut the computation cost further, the HT could be improved by using parallel computing techniques or by using the random Hough transform (RHT) method [23].

#### 4.2.2. SNR Analysis

**Figure 11:**
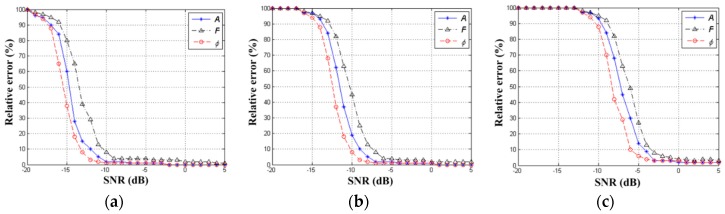
Relative errors versus different Set the signal-to-noise ratios (SNRs). (**a**) Result of T1. (**b**) Result of T2. (**c**) Result of T3.

Since HT is a cumulative process, the proposed algorithm has ideal anti-noise property. To analyze the influence of noise on the relative error of MM target parameters, we set target T1, T2 and T3 (their parameter settings are consistent with Section 4.1) in the scene. After performing 800 Monte-Carlo detection experiments where SNRs vary from −20 to 5 dB, the relative error curves are illustrated in Figure 11. Figure 11a–c are the relative error result of T1, T2 and T3, respectively. We find all the parameter relative errors decrease with the increase of SNR. When SNR>−2 dB, the relative errors of parameters of T1, T2 and T3 become small (relative errors of A, F and φ are <5%). When SNR<−10 dB, resolution of the TF distribution is badly impaired, resulting in the accuracy of parameter estimation becomes worse. From the results of Figure 11, we can see the detection performance of T1 is better than T2, and the detection performance of T2 is better than T3. This is because the algorithm in this paper is a sequential detection process (i.e., T1 must be detected before T2 and T3 can be detected one after another). The detection accuracy of the former component seriously affects the detection result of the latter component. Only by accurately estimating the previous component can the component be eliminated more thoroughly. Moreover, when the previous component is eliminated one after another, the relative noise of the component behind will be larger. Therefore, the detection performance of the weak components behind are gradually decreasing.

#### 4.2.3. The Ideal Peak Search Performance

The simulation results in Section 4.1 show that the position of the ideal peak obtained is basically consistent with its real position, which indicates that the search method we use is almost not affected by false peaks. The reason is as follows. The features of the false peaks generated after HT are related to the number of sinusoidal curves, noise intensity and parameter search step size. When the number of sinusoidal curves gets smaller and noise intensity becomes lower, the false peaks become more intensely distributed and their values become smaller. Likewise, when the parameter search size gets larger, the false peaks also become more intensely distributed and their values get smaller. The proposed algorithm only deals with the TF curve with the strongest energy (There is only one such curve.), and the parameter search step size we set is relatively large ($\Delta f_{1}=\Delta f_{2}=1\mathrm{Hz}$, $\Delta \varphi =1^{{}^{\circ}}$), so the false peaks are intensely distributed and their values is much smaller than that of the ideal peak. Therefore, the maximum search method can achieve good results. When SNR is too small, the results of Monte-Carlo simulation in Section 4.2.2 indicate that the estimation accuracy of the parameters is seriously affected. Influenced by the strong noise, part of the values of the false peaks after HT exceeds that of the ideal peak. At this time, the maximum search method may fail to locate the ideal peak.

#### 4.2.4. The Maximum Number of Targets and Maximum Dynamic Range of the Method

In order to study the maximum number of targets and the maximum dynamic range of the method, we assume that the azimuth signal contains M targets, their reflection coefficients are $\sigma_{1},\sigma_{2},...,\sigma_{M}$ from large to small, and the energy of the noise signal mixed in the azimuth signal is $N_{0}$. In this paper, the definition of the *n*th target’s relative SNR is defined as $\sigma_{n}^{2}/N_{0}$. The method of eliminating strong target step by step is to reduce the influence of strong targets on weak targets. However, in the process of eliminating strong targets, the energy of the noise cannot be eliminated (that is, the energy of signal noise is unchanged), so the relative SNR of weaker targets will continue to decrease. By detecting randomly generated MM targets with different reflection coefficients many times, we found that a target can be detected accurately only when the relative SNR of the target is greater than critical SNR($SNR_{\min}$)($SNR_{\min}$ is usually −10 dB to −9 dB). If the relative SNR of a target is less than $SNR_{\min}$, we cannot accurately obtain the MM parameters of the target, then there will be a strong residual after eliminating it, so that the subsequent weak targets cannot be detected. Therefore, when $\sigma_{i}^{2}/N_{0}=SNR_{\min}$, then the minimum reflection coefficient of targets is $\sigma_{i}$ and the maximum number of targets is i. Due to the requirements of $SNR_{\min}$ in this algorithm are very small, the algorithm has good detection ability for multi-MM targets with large dynamic reflection coefficients.

#### 4.2.5. Limitations of the Algorithm

As is previously proved, when there are several MM targets with large different amplitudes, the algorithm in this paper can achieve good results. However, in the case of two or more MM targets with equal or similar amplitudes, this algorithm will not work. This is because TF curve extraction in this algorithm is based on the maximal amplitude of TF distribution. An error may occur in extracting TF curve when there are multiple targets with similar energy. Since the algorithms proposed in [10,15] have realized the detection of multiple MM targets with similar energy, the application range of our algorithm can be extended when it is combined with them.

### 4.3. Field Experiment

**Figure 12:**
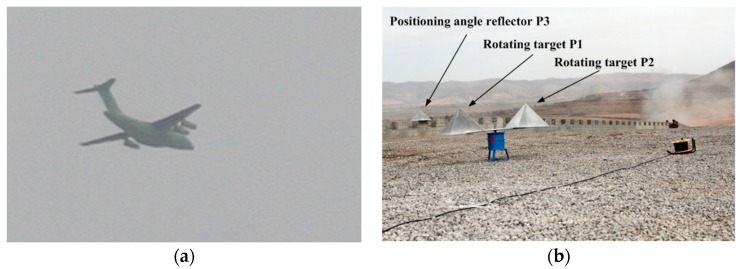
On-site shooting. (**a**) Yun-8 aircraft; (**b**) Angle reflectors.

**Figure 13:**
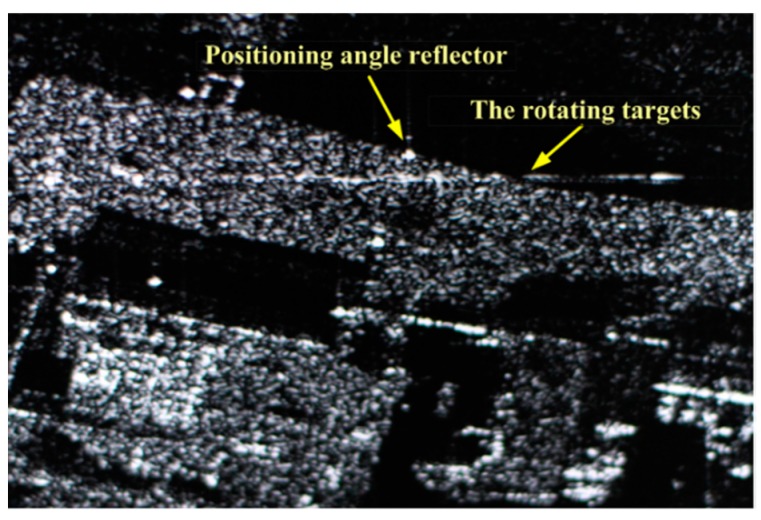
SAR image result of the scene.

In this part, we conduct experiments to check into our algorithm using X-band SAR. The SAR is equipped on Yun-8 aircraft (Figure 12a) with a flying altitude of 6000 m and a flying velocity of 168 m/s. The carrier frequency of SAR is 9.8 GHz, the PRF is 400 Hz, and the synthetic aperture time is 1.5 s. In the experiment, SAR operates in side-view mode. A symmetrical rotating angle reflector P1, P2 and a fixed angle reflector P3 are placed in the scene, wherein all reflectors are of size 0.25 m×0.25 m×0.18 m, the angle reflectors P1, P3 are made of aluminum, and the angle reflector P2 is made of reinforced plastics. The effective radii of the two rotating reflectors are both 0.3 m and their rotational frequencies are 1.5 Hz. The angle reflector P3 serves as a positioning angle reflector, and the angle reflectors P1 and P2 are the MM targets to be detected. Although P1 and P2 are of the same size, the reflection coefficient of P1 is stronger than that of P2. After SAR gets the echo, we use R-D algorithm for imaging. The on-site shooting of the scene is shown in Figure 12b and the imaging result of SAR is illustrated in Figure 13. We can see that the positioning angle reflector focuses well, while the rotating targets produce azimuth defocusing, which is caused by the micro-Doppler effect.

**Figure 14:**
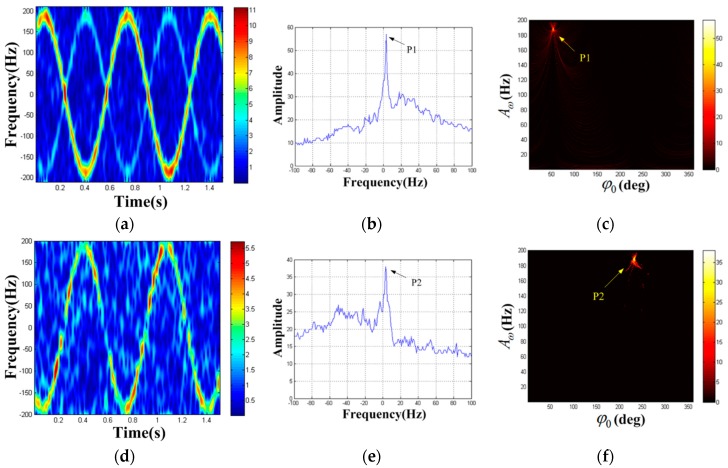
Simulation detection result of rotating angle reflectors. (**a**) TF distribution result of the azimuth echo; (**b**) $F_{1}$-domain; (**c**) $\left\{A_{1},\varphi_{1}\right\}$ domain when $F_{1}=1\mathrm{Hz}$; (**d**) TF distribution result of the residual signal; (**e**) $F_{2}$-domain; (**f**) $\left\{A_{2},\varphi_{2}\right\}$ domain when $F_{2}=0$.

Before processing the real echo data, we use the computer to obtain the simulation echo data of P1 and P2 in the scene. Then the azimuth echo of the rotation targets is extracted, and the rotation frequency can be estimated to be 1.5 Hz by autocorrelation method. Next, we use the algorithm to process the simulation echo signal and the results are shown in Figure 14. Figure 14a is the STFT result of the azimuth echo (the Kaiser window’s width is 37), Figure 14b,c is the HT result of the strongest component TF curve, Figure 14d is the STFT result of the residual azimuth echo, and Figure 14e,f is the HT result of the residual signal. Therefore, the estimated values of MM parameters of P1 and P2 can be obtained as $\left({\hat{A}}_{\omega }^{\left(1\right)},{\hat{\varphi }}_{0}^{\left(1\right)},{\hat{F}}^{\left(1\right)}\right)=\left(188\;\mathrm{Hz},52^{{}^{\circ}},1\;\mathrm{Hz}\right)$ and $\left({\hat{A}}_{\omega }^{\left(2\right)},{\hat{\varphi }}_{0}^{\left(2\right)},{\hat{F}}^{\left(2\right)}\right)=\left(188\;\mathrm{Hz},232^{{}^{\circ}},2\;\mathrm{Hz}\right)$ respectively.

**Figure 15:**
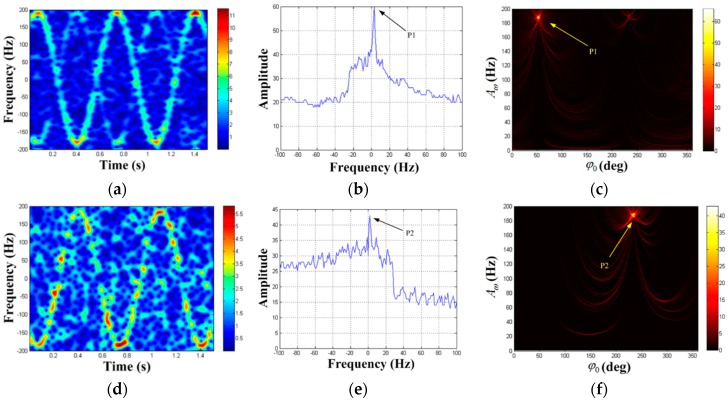
Real echo detection result of rotating angle reflectors. (**a**) TF distribution result of the azimuth echo; (**b**) $F_{1}$-domain; (**c**) $\left\{A_{1},\varphi_{1}\right\}$ domain when $F_{1}=1\mathrm{Hz}$; (**d**) TF distribution result of the residual signal; (**e**) $F_{2}$-domain; (**f**) $\left\{A_{2},\varphi_{2}\right\}$ domain when $F_{2}=0$.

The real echo data processing is carried out below. Initially, we extract the azimuth echo where rotating targets exist and obtain the rotational frequency as 1.5 Hz. The TF distribution of the azimuth echo acquired by STFT (the Kaiser window’s width is 37) is shown in Figure 15a. According to the result, the component of P1 is apparent while the component of P2 is hardly visible. Then, the strongest TF curve extracted in Figure 14a is processed by HT, and the results can be seen in Figure 15b,c. The existence of a peak can be clearly seen in HT results. Hence, the parameter estimation of P1 can be obtained as $\left({\hat{A}}_{\omega }^{\left(1\right)},{\hat{\varphi }}_{0}^{\left(1\right)},{\hat{F}}^{\left(1\right)}\right)=\left(189\;\mathrm{Hz},53^{{}^{\circ}},1\;\mathrm{Hz}\right)$. Next, the component of P1 is removed from the original azimuth echo. After processing the residual signal by autocorrelation method, we find there is also a periodic signal with frequency of 1.5Hz in the residual signal. The residual signal is processed by the STFT (the Kaiser window’s width is 37) and the TF distribution can be seen in Figure 15d. The component of P1 has been removed, and the component of P2 is highlighted. Then, we perform HT on the TF curve extracted in Figure 14d, and the results can be seen in Figure 15e,f. Thus, the parameter estimation of P2 is $\left({\hat{A}}_{\omega }^{\left(2\right)},{\hat{\varphi }}_{0}^{\left(2\right)},{\hat{F}}^{\left(2\right)}\right)=\left(188\;\mathrm{Hz},232^{{}^{\circ}},0\right)$. According to $A_{\omega }=4\pi r_{0}f_{a}/\lambda$, we can get the rotational radii of P1 and P2 as 0.307 m and 0.305 m respectively with the estimated values of the rotational frequency and the maximal m-D frequency. Moreover, the initial phase difference between P1 and P2 is $179^{{}^{\circ}}$, which generally accords with the size of the rotating angle reflectors. It is shown that both rotating targets with large reflection coefficient differences are detected successfully and the parameters are estimated accurately. Again, we verify the correctness of the proposed algorithm.

## 5. Conclusions

In this study, we propose a detection algorithm for MM target with large dynamic reflection coefficients. Based on TF analysis, the algorithm uses HT to detect the strongest MM component first, and removes this component from the original azimuth echo. Then, the process is repeated, and the strong target components are removed one by one until all the MM targets are detected. Simulations and field experiments demonstrate the proposed algorithm is capable of detecting MM targets accurately, even though the reflection coefficients of the targets differ greatly. Monte-Carlo experiments indicate that the proposed algorithm has ideal anti-noise property. In addition, the computational speed of our algorithm can be improved by certain methods (for example, RHT and parallel computing), making real-time MM target detection possible. Therefore, our algorithm has strong practical value.

## Figures and Tables

**Table 1:** Target parameter setting.

Target	Reflection Coefficient	Rotational Frequency/Hz	Effective Radius/m	Initial Phase	Rotation Center Coordinate
T1	2.4	2	0.15	120°	(−53 m,8000 m,0)
T2	1.2	1.5	0.16	60°	(15 m,8000 m,0)
T3	0.7	1.2	0.18	30°	(30 m,8000 m,0)

**Table 2:** Theoretical values and estimated values of the parameters.

Targets	Parameters	Theoretical Values	Estimated Values	Relative Errors
T1	$f_{a}^{\left(1\right)}$	2 Hz	2 Hz	0
	$A_{\omega }^{\left(1\right)}$	125.6 Hz	125 Hz	0.48%
	$\varphi_{0}^{\left(1\right)}$	120°	120°	0
	$F^{\left(1\right)}$	−70.5 Hz	−69 Hz	2.1%
T2	$f_{a}^{\left(2\right)}$	1.5 Hz	1.5 Hz	0
	$A_{\omega }^{\left(2\right)}$	100.5 Hz	101 Hz	0.49%
	$\varphi_{0}^{\left(2\right)}$	60°	60°	0
	$F^{\left(2\right)}$	20 Hz	21 Hz	5%
T3	$f_{a}^{\left(3\right)}$	1.2 Hz	1.2 Hz	0
	$A_{\omega }^{\left(3\right)}$	90.4 Hz	89 Hz	1.5%
	$\varphi_{0}^{\left(3\right)}$	30°	30°	3.3%
	$F^{\left(3\right)}$	40 Hz	43 Hz	7.5%
